# Variation in cranial and caudal keel bone composition and its association with keel damage severity in laying hens

**DOI:** 10.1016/j.psj.2026.106859

**Published:** 2026-03-24

**Authors:** Khrystyna Kurta, Moh Sallam, Lina Elmersson, Tattiana Enrich-Essvein, Sky Vias-Valera, Stefan Gunnarsson, Helena Wall, Ian C. Dunn, Dirk-Jan de Koning, Martin Johnsson, Alejandro B. Rodriguez-Navarro

**Affiliations:** a Department of Animal Biosciences, Swedish University of Agricultural Sciences, Uppsala, Sweden; b Center for Quantitative Genetics and Genomics, Aarhus University, Aarhus, Denmark; c Department of Applied Animal Science and Welfare, Swedish University of Agricultural Sciences, Uppsala and Skara, Sweden; d Departamento de Mineralogia y Petrologia, Universidad de Granada, Granada, Spain; e Roslin Institute, University of Edinburgh, Easter Bush, Midlothian, Scotland, UK

**Keywords:** Keel bone damage, Bone mineralization, Bone composition, Laying hens, Welfare

## Abstract

Keel bone damage is prevalent in commercial laying hen flocks and is commonly associated with reduced mineralization of this bone. The aim of this study was to characterize variation in cranial and caudal keel bone composition and to examine its association with keel damage severity in Bovans Brown laying hens. The cranial region of the keel was significantly more mineralized than the caudal region (mineral content: 53 ± 4 % vs. 46 ± 5 %, *P* < 0.05). Low to moderate correlations (*r* = –0.27 to 0.55, *P* < 0.05) were observed between keel bone chemical composition and radiographic measurements of keel shape (cranial depth, keel length, and mid-depth), suggesting that mineral matrix composition contributes to keel structural development. Furthermore, increasing keel damage severity, assessed by callus extent and size, was associated with significant reductions in lipid content (median: 0.06 to 0.03), mineral content (56 % to 52 %), and the mineral-to-organic ratio (1.7 to 1.4) in the cranial keel cortex (*P* < 0.05). These findings indicate that reduced keel bone mineralization was associated with increased susceptibility to deformation and fracture. Overall, this study enhances our understanding of keel bone biology in laying hens and identifies compositional factors that may contribute to susceptibility to keel damage and fractures.

## Introduction

The keel bone, or carina, of birds is the large flat extension of the breastbone that serves as the attachment for breast muscles and mineralizes by endochondral ossification (Gibson et al., 1995). During rearing, the hen’s skeleton grows rapidly until reaching sexual maturity at 16 weeks of age, ceasing further development when reaching a peak in egg laying at about 24-28 weeks of age, as commonly observed in the modern commercial flocks (Bain et al., 2016). However, at this age the keel bone is poorly mineralized at the caudal part, and mineralization may continue until hens are 40 weeks old (Buckner et al., 1948; Gretarsson et al., 2025) or more, and this process may compete for calcium with eggshell formation, which also demands a high and continuous supply of calcium, reviewed by (Nys and Le Roy, 2018). The keel bone calcification is uneven over time as the mineralization front progresses from the cranial to the caudal end (Benavides-Reyes et al., 2021; Gibson et al., 1984). Thus, the late and incomplete keel mineralization, makes it more vulnerable to deformation and fracture resulting in a high percentage of birds having keel bone damage (Eusemann et al., 2018).

Keel bone damage is prevalent in commercial laying hen flocks (Rufener and Makagon, 2020; Sallam et al., 2024a; Thøfner et al., 2021) and is normally associated with a deficient mineralization of this bone (Gebhardt-Henrich et al., 2017; Maidin et al., 2024). Gebhardt-Henrich et al. (2017) found that mineral and calcium content of the keel bone are associated with the occurrence of keel bone fractures in laying hens. Most fractures occur in the caudal part of the keel and result in callus formation. Deformations where the keel deviates from a flat sagittal plane in the middle of the chest, taking an S-shape are also common. Keel fracture is associated with deviation in keel shape though the direction of causality is not clear (Maidin et al., 2024; Sallam et al., 2024a). Structural characteristics of the keel bone, including cortical density and cortical thickness measured using quantitative computed tomography (QCT), have also been investigated in relation to keel bone deformities and damage (Regmi et al., 2016).

Keel bone fractures are associated with pain and represent a major welfare problem (Riber et al., 2018). Behavioral studies find that hens with fractured keels have reduced mobility, and that that analgesics improve mobility, suggesting pain (Montalcini et al., 2024; Nasr et al., 2012, 2023; Rufener et al., 2019a). Hens with fractured keels show endocrine and neural correlates of inflammation and stress (Armstrong et al., 2020; Nasr et al., 2012; Wei et al., 2019). Keel bone damage may also cause economic losses for the producers in the form of production losses (Nasr et al., 2012; Rufener et al., 2019b; Wei et al., 2020). The prevalence of keel bone damage is expected to increase with industry shift toward cage-free housing, as these open systems are associated with a significantly higher incidence of fractures compared to furnished cages, reviewed by (Hardin et al., 2019).

Keel bone quality and susceptibility to damage are influenced by a wide array of factors including genetics, nutrition, age and housing (Hardin et al., 2019; Marggraff et al., 2024). Several studies have found low to moderate heritability for keel quality and shape traits (Duenk et al., 2025; Maidin et al., 2024; Sallam et al., 2025a). Comparisons between fractured and unfractured keel bones also suggest that there are differences in mineral composition and structure, including lower density and bone volume in fractured keels (Wei et al., 2021, 2023). On the contrary, other studies have found limited association between keel bone traits and fractures (Gebhardt-Henrich et al., 2017), and an inverse relationship where callus formation leads to higher density in fractured keels (Maidin et al., 2024; Sallam et al., 2025a).

In the present study, we first aimed to determine whether the mineralization and chemical composition of the keel bone differ between the cranial and caudal anatomical regions. Second, we investigated how keel bone density, geometry and mineralization are related to the severity of keel bone damage. Third, we studied how keel mineralization is related to the mineralization of other parts of the skeleton using the tibia as representative bone. This study can help to understand the development of keel bone damage by identifying how different bone morphological, structural or chemical characteristics might correlate to keel bone damage.

## Materials and methods

### Ethics statement

The animal study was approved by Gothenburg Local Ethics Committee of the Swedish National Board for Laboratory Animals. The study was conducted in accordance with the local legislation and institutional requirements (Reference 5.8.18-16645/2020).

### Birds housing and management

The analyses was based on Bovans Brown laying hens that were a part of the previous study that was carried out at a commercial farm in Sweden. The birds were non-beak-trimmed and arrived at the facility at 16 weeks of age. Throughout the production cycle, hens were housed in a multi-tier aviary system at a stocking density of nine hens per m^2^ (calculated on available area). The lighting program was according to the manual of the hybrid, and the birds had *ad libitum* access to standard commercial food and water, and wood shavings were used as litter material.

### Experimental group and sampling

A subset of hens within the flock was separated by a temporary mesh wire wall. From this group, 220 hens were randomly selected and individually identified using plastic yellow wing tags (48 mm × 42 mm). These hens were analyzed in the previous study (Sallam et al., 2024a) for keel bone condition and damage scores and were used in the present study for the bone composition analyses.

### Slaughter and tissue collection

At the end of the production cycle (74 weeks of age) these birds were collected and retained for final weighing and clinical examination. They were euthanized via percussive stunning to the head followed by immediate cervical dislocation and exsanguination. The birds were individually labeled, sealed into plastic bags, and stored at −20°C at the Skara research station, Swedish University of Agricultural Sciences, Sweden. Thereafter, a trained team dissected the focal birds post-mortem and extracted the right and left tibiotarsal bone and the keel bone, placing them in labeled plastic bags for radiographic examination and scoring.

### Dissected keel damage measurements

**Fig. 1:**
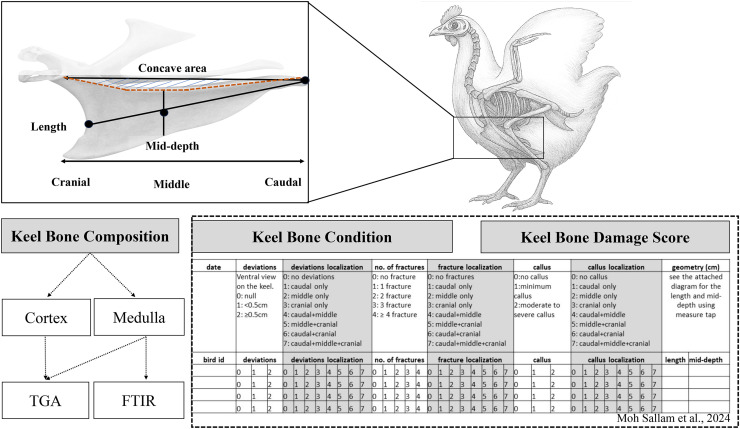
Schematic overview of the experimental design for the analysis of keel bone quality. Measurements of bone chemical composition traits were obtained as part of the present study. Data on keel bone condition deviations, fractures, and callus formation) and damage scores were available from the previous study (Sallam et al., 2024a).

Keel bone condition (or shape) and damage scores were available from a previous study (Sallam et al., 2024a). A detailed description of the method and scoring protocol can be found in (Sallam et al., 2024a). Briefly, the dissected keel bones were scored by two experienced veterinarians based on a protocol adapted from (Thøfner et al., 2021). Damages were assessed in terms of deviations, fractures and callus formation in the cranial, middle, and caudal part of the keel bone (Fig. 1). In addition, the following keel bone condition parameters were registered: keel bone length, keel bone mid-depth, keel bone cranial depth, and radiographic keel density (radiographic optical density of the cranial part of the keel as explained in (Sallam et al., 2024a; Fig. 1). In the present study, these results were then linked to the chemical composition measurements conducted postmortem on the same keel bone samples.

### Keel and tibial bone composition analyses

For each bird, the dissected two tibiae and keel bone were analyzed by thermogravimetry (TGA), and Fourier transform infrared spectroscopy (FTIR). The cortical and medullary bone from the tibia mid-diaphysis were manually separated using a scalpel. In keels, two different regions (cranial and caudal end) were sampled. For TGA analyses, we could not separate enough medullary bone so a piece of keel containing both tissues was measured. For FTIR, we could analyze the cortical and medullar tissue of keels by separating the two walls and measuring the cortical and medullary tissues on the inner surfaces. Medullary bone was measured directly on the inner wall surface and the cortical bone after removing the medullary bone by grinding until reaching the cortical bone.

The percentage of water, organic matter, carbonate and mineral content, and carbonate/mineral ratio in each bone sample were determined by thermogravimetry (TGA). Powdered bones were treated by heating for 1 h at different temperatures (200, 600 and 800 °C) in an RWF 1100 furnace (Carbolite, UK) and weighed to determine the weight percentage of each chemical component. The following TGA traits were analyzed in this study: mineral content, organic content, mineral/organic ratio, carbonate content, carbonate/mineral ratio.

The chemical composition of bone tissues (cortical and medullary bone) was analyzed by infrared spectroscopy using a FTIR spectrometer JASCO 6200 equipped with a diamond attenuated total reflection (ATR) accessory (ATR Pro ONE, JASCO). The detail description of the methodology was described in (Rodriguez-Navarro et al., 2018). Different chemical components and compositional parameters (e.g., degree of mineralization; PO_4_/Amide I) were determined from main IR bands (Phosphate v1, v3 PO_4_: 900–1200 cm^-1^; Amide I: 1590–1710 cm^-1^; Carbonate; 1410 cm^-1^; Lipids: 1720 cm^-1^). The following FITR traits has been analyzed in the present study: relative amount of carbonate in bone mineral to organic matrix (CO_3_1415/AmideI), total amount of carbonate in bone mineral (MinCO_3_1415); the amount of carbonate substituted in the mineral as the ratio of CO_3_ (870 cm^−1^) to the main phosphate (900 to 1200 cm^−1^) band area; relative amount of mineral to organic matrix PO_4_/AmideI; ratio of Amide II relative to organic matter Amide I (AmideI/AmideII); mature and immature collagen or collagen cross-linking (LNK 1660/1690 ratio); carbonyl group from the lipid (Lipids).

### Statistical analyses

The summary statistics for all data was computed using R base packages (R Core Team, 2019). For keel compositional traits, we compared the cranial and caudal regions separately within the cortex and medulla to identify potential spatial differences in bone quality (Table S1). For tibiae, the mineral composition data from the left and right tibiae were averaged (Table S2). These mean values were then utilized for all subsequent statistical analyses and correlations. Additionally, 95 % confidence intervals (CI) were calculated for all correlations coefficients. Mean values and standard errors were visualized using bar plots, and statistical comparisons were performed using the Wilcoxon rank-sum test.

Pearson correlation was used to estimate the correlation coefficients between continuous traits such as keel morphology and compositional parameters. Spearman rank correlation was used to evaluate associations between keel bone composition traits (continuous) and keel damage traits (ordinal categorical; scored 0 to 4), as it does not assume normal distribution and accounts for ordinal scaling. All correlation coefficients and corresponding significance values were computed using the cor.test() function in R.

## Results

### Regional comparison of keel bone composition traits

We investigated variation in the keel degree of mineralization and other chemical composition traits measured using TGA and FTIR analyses to assess whether the composition of the keel bone differs between the cranial and caudal anatomical regions. Summary statistics for these traits, along with the number of birds remaining after filtering for non-missing values, are provided in Supplementary Table S1.

**Fig. 2:**
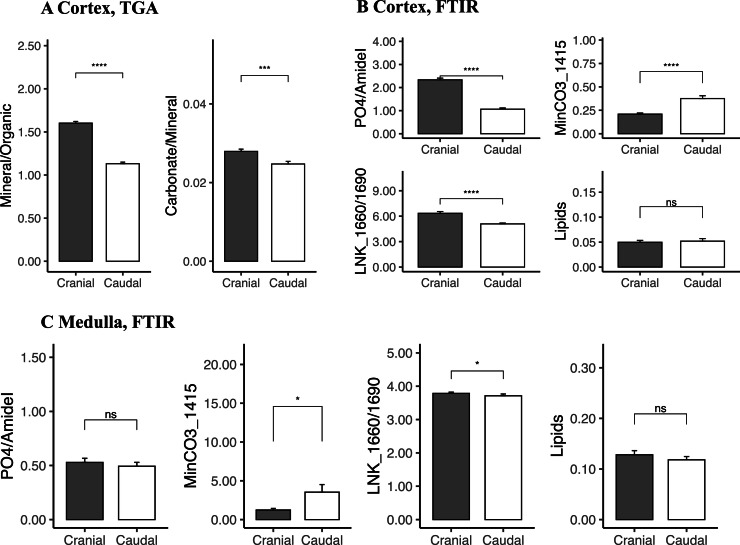
Mean keel bone composition traits determined using TGA (A) and FTIR (B, C) measured at the cranial and caudal regions in the cortex (A and B) and in the medulla (C). Bar heights represent group means; error bars indicate standard errors. Statistical significance was assessed using the Wilcoxon test: *P < 0.05, **P < 0.01, ****P < 0.001, ns stands for not significant.

Keel bone at the cranial region was significantly more mineralized (mineral content: 53 ± 4 %) than the keel at the caudal end (mineral content: 46 ± 5 %) as the mineralization starts at the cranial region and progresses with time toward the caudal end (Wilcoxon test, *P* < 0.001, Fig. 2a, Table S1). In both keel regions, the mineral content showed a relatively high variability (CV = 10 %) compared to other bone (tibia cortex; CV= 2 %). Also, other compositional parameters determined by TGA (mineral/organic ratio, carbonate to mineral ratio) were significantly higher in the cranial than in the caudal region. There were significant differences between cranial and caudal keel regions in the degree of mineralization and other composition traits measured in the keel cortex by FTIR. For example, the keel cortex degree of mineralization (PO_4_/AmideI), also showed significant differences between the different regions (cranial vs caudal), confirming that the caudal end is less mineralized than the cranial region.

Other FTIR-derived compositional parameters (i.e., amount of lipids) did not show any significant differences among the regions sampled (cranial vs caudal; Fig. 2b). In the keel medullary bone, only one parameter, associated to collagen cross-linking (LNK 1660/1690 ratio), showed significant differences between cranial and caudal keel parts, suggesting that medullary bone has a homogenous distribution and composition along the keel medulla cavity (Fig. 2c). The lack of compositional variation in medullary bone could be due to its continuous renovation due to its faster turnover rate which is coupled with the daily egg cycle.

### Relationship between keel composition and keel condition traits

**Fig. 3:**
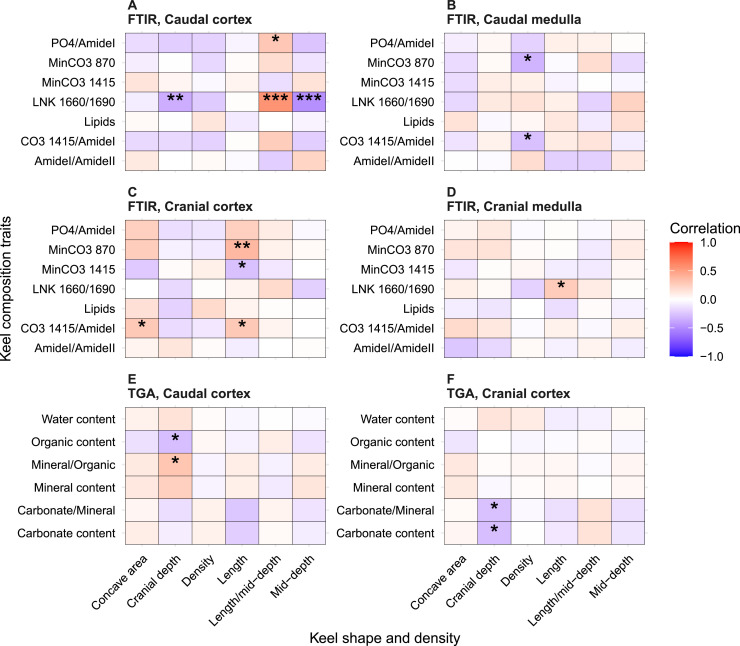
Pearson correlations between keel bone composition traits and keel shape and density. Statistical significance is indicated as follows: *P < 0.05, **P < 0.01, ***P < 0.001.

We examined dissected keel bones from laying hens to assess the relationship between keel condition (shape and density) and keel compositional traits (Fig. 3).

Keel cranial depth showed significant correlation with several bone composition traits in both cranial and caudal parts of the keel (Fig. 3). Specifically, the keel cranial depth was negatively correlated to TGA carbonate content (*r* = –0.28, 95 % CI −0.5 to −0.02, *P* < 0.05) and carbonate/mineral ratio (*r* = –0.27, CI −0.5 to −0.02, *P* < 0.05) at the cranial cortex region, and positively correlated to the degree of mineralization (mineral/organic ratio; *r* = 0.30, CI 0.05 to 0.52, *P* < 0.05) in the caudal cortex (Fig. 3).

Keel length showed significant correlations with FTIR-metrics in the cranial keel cortex, including positive coefficients with MinCO_3_870 (*r* = 0.37, CI 0.12 to 0.57, *P* < 0.01) and CO_3_1415/AmideI (*r* = 0.28, CI 0.03 to 0.5, *P* < 0.05), while negative with MinCO_3_1415 (*r* = –0.27, CI −0.49 to −0.01, *P* < 0.05). The ratio CO_3_1415/AmideI in the cranial cortex, which represents a measurement of the degree of mineralization, was significantly and positively correlated with the keel concave area (*r* = 0.28, CI 0.02 to 0.5, *P* < 0.05). In the cranial keel medullary region, the parameter related to crosslinking of collagen (LNK 1660/1690) also showed a significant and positive correlation with keel length (*r* = 0.27, CI 0.01 to 0.49, *P* < 0.05).

In the caudal keel cortex, collagen crosslinking (LNK 166/1690) exhibited the strongest significant and negative correlation with keel mid-depth (*r* = –0.48, CI −0.66 to −0.25, *P* < 0.001), positive correlation with the ratio of keel length to mid-depth (*r* = 0.55, CI 0.35 to 0.71, *P* < 0.001), and a lower negative correlation with keel cranial depth (*r* = –0.35, CI −0.56 to −0.11, *P* < 0.01). The PO_4_/Amide I (a measurement of the degree of mineralization) was positively correlated with the length-to-mid-depth ratio (*r* = 0.29, CI 0.03 to 0.51, *P* < 0.05). In the caudal keel medullary region, MinCO_3_870 (*r* = –0.32, CI −0.55 to −0.05, *P* < 0.05) and CO_3_1415/Amide I (*r* = –0.27, CI −0.5 to −0.01, *P* < 0.05) were negatively correlated to keel bone density.

### Relationship between keel composition and keel damage categories

We utilized post-mortem keel bone damage data from previous studies (Sallam et al., 2024a) and examined their relationships with keel bone composition traits measured in both the cortex and medulla in the cranial and caudal regions of the keel.

**Fig. 4:**
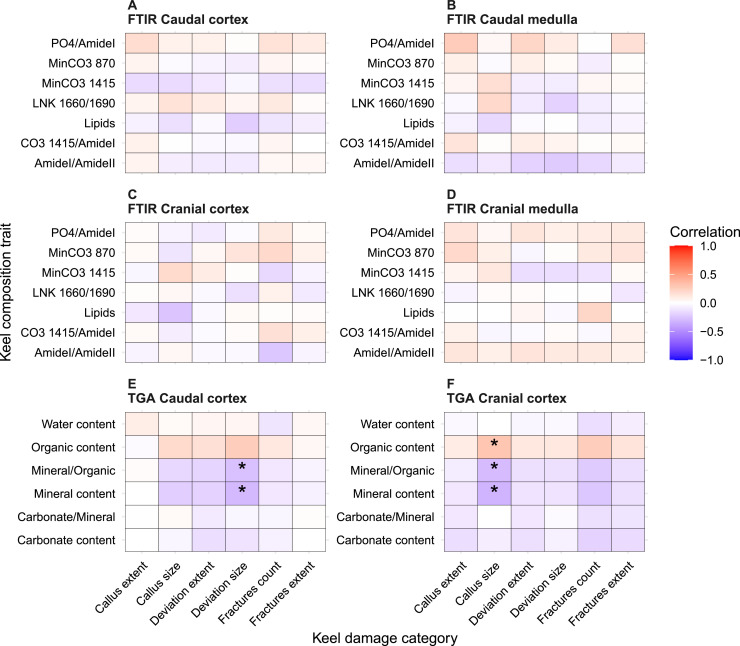
Spearman correlations between keel bone composition traits and keel damage category traits. Statistical significance is indicated as follows: *P < 0.05, **P < 0.01, ***P < 0.001.

Callus size was significantly negatively correlated with mineral content and the mineral/organic ratio (*r* = –0.3, CI −0.53 to −0.06, *P* < 0.05) and positively correlated with organic content (*r* = 0.3, CI 0.04 to 0.52, *P* < 0.05) in the cranial cortex. Furthermore, keel deviation size was negatively correlated with mineral content and the mineral/organic ratio in the keel caudal region (*r* = –0.3, CI −0.5 to −0.04, *P* < 0.05; Fig. 4).

### Variation in keel bone composition per keel damage severity

**Fig. 5:**
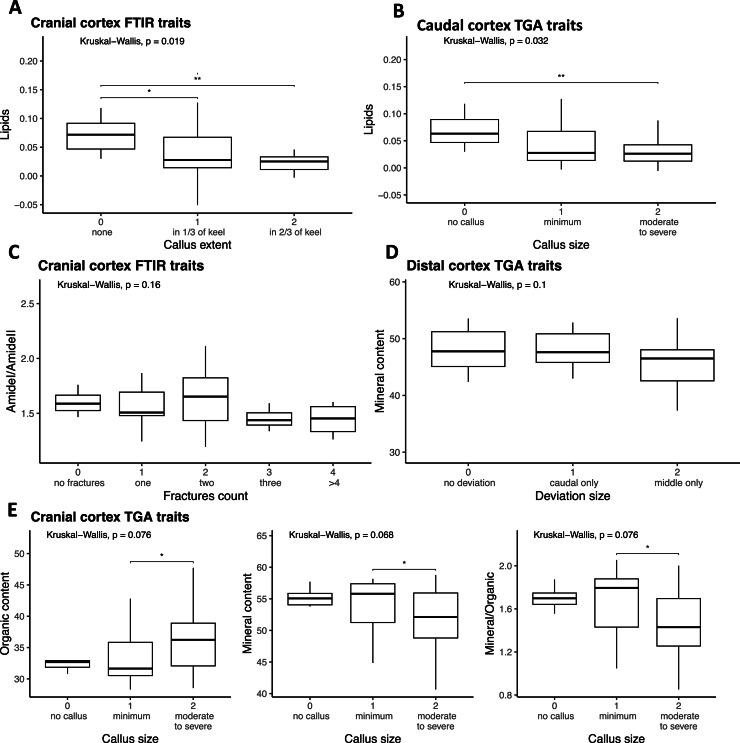
Distribution of keel bone composition traits by keel damage categories. Statistical differences were assessed using the Kruskal–Wallis test. Statistical significance is indicated as follows: *P < 0.05, **P < 0.01, ***P < 0.001.

Next, we explored group-wise differences in keel composition traits across keel damage categories (Table S3, Fig. 5). Only traits that showed significant correlations with keel damage parameters from the section above were included in this analysis.

Significant associations between keel damage and measured bone composition traits were observed in the keel cortex, rather than in the medulla. In particular, the Kruskal–Wallis test revealed a significant difference in lipid content in the cranial cortex of the keel among birds with varying callus extent categories (*P* < 0.05; *η*^2^ = 0.15). Birds with callus covering one-third or half of the keel exhibited lower lipid levels (median = 0.03) compared to those without callus (median = 0.07). Similarly, lipid levels in the keel cortex were lower in birds with moderate to severe callus (lipids content median = 0.03; *η*^2^ = 0.07) than in birds without callus (median = 0.06). Additionally, birds with moderate to severe callus showed higher organic content (36 %; *η*^2^ = 0.06) and lower mineral content (52 %; *η*^2^ = 0.07) compared to birds with minimal callus in the cranial cortex (organic: 32 %, mineral: 56 %). Lower Amide I/Amide II ratios that are related to collagen orientation (drop from 1.59 to 1.45; *η*^2^ = 0.05) were observed with increasing fracture counts in the cranial cortex, and a downward trend in mineral content (from 48 % to 45 %; *η*^2^ = 0.05) was associated with larger keel deviation size in caudal cortex; however, neither of these differences reached statistical significance of *P* < 0.05 (Fig. 5, Table S3). These data indicate that reduced keel bone mineralization is associated with increased susceptibility to deformation and fracture.

### Association between keel and tibia composition traits

Potential associations between bone composition traits of the keel and tibia using both TGA and FTIR measurements were next investigated. Statistically significant correlations were detected between several traits across the two bones.

Within the TGA dataset measured in the cortical bone of the keel and tibia, correlations were generally modest, ranging from –0.57 to –0.33. The strongest statistically significant associations were observed between water content in the caudal keel cortex and tibial cortical TGA traits, such as organic (*r* = 0.44, CI 0.33 to 0.54, *P* < 0.001), water (*r* = –0.57, CI −0.65 to −0.47, *P* < 0.001), and carbonate contents (*r* = –0.45, CI −0.55 to −0.33, *P* < 0.001), carbonate/mineral (*r* = –0.46, CI −0.56 to −0.35, *P* < 0.001), and mineral/organic ratios (*r* = –0.34, CI −0.46 to −0.22, *P* < 0.001; Fig. S1).

Withing the FTIR dataset, significant correlation coefficients were generally low to moderate, ranging from –0.3 to 0.39. The most robust and consistent associations between keel and tibial compositional traits were observed in the medullary bone. Specifically, the strongest positive correlations (*P* < 0.05) were observed for CO_3_1415/AmideI (*r* = 0.39, CI 0.26 to 0.5, *P* < 0.001) and lipid content (*r* = 0.37, CI 0.24 to 0.48, *P* < 0.001); these relationships were consistent across both cranial and caudal regions of the keel. In contrast, the cortical bone exhibited a moderate negative correlation (*r* ≈ –0.3, CI −0.41 to −0.18, *P* < 0.001) between keel lipid content (cranial and caudal) and tibial MinCO_3_1415 (Fig. S2). These associations may reflect changes in bone mineral composition during mineralization and maturation.

## Discussion

### Keel bone composition differs between cranial and caudal regions

In this study, we examined regional variation in keel bone composition and mineralization in laying hens by analyzing samples from two anatomically distinct areas: the cranial and caudal regions of the keel. This study shows that keel bone mineralization remains lower than that of the tibial cortical bone even at the end of the laying cycle (74 weeks of age), and that mineralization is significantly lower in the caudal region compared with the cranial region. In addition, significant compositional differences between the cranial and caudal regions were observed in cortex, but not in the medullary bone. The cranial cortex exhibited consistently higher mineralization than the caudal cortex, as reflected by significant differences across both TGA (mineral content, mineral to organic ratio) and FTIR parameters (PO_4_/AmideI, CO_3_1415/AmideI). Our results align with previous research demonstrating a cranial-to-caudal decline in mineralization along the keel (Benavides-Reyes et al., 2021). This gradient in terms of degree of mineralization across the keel length appears to be maintained even in older hens and may affect keel’s mechanical properties, presumably making it more prone to deformation and fractures particularly at the mineralization front close to caudal region. The keel compositional profile found in this study suggests that the cranial region has a denser and more mineral-rich cortical matrix, whereas the caudal cortex retains a comparatively higher organic fraction and lower mineral density. These factors may contribute to the increased susceptibility to fractures in the caudal region or its deformation due to reduced mineral density and structural integrity (Gretarsson et al., 2024; Li et al., 2024; Toscano et al., 2020).

### Association between keel bone composition and keel shape

The keel bone shape, size, and density are crucial for its mechanical function (Habig et al., 2021; Maidin et al., 2024) and easily measurable by X-ray imaging in live birds (Sallam et al., 2024b). The observed correlations between keel bone chemical composition and radiographic image measurements of keel shape (cranial depth, keel length and mid-depth) in the present study, suggest that mineral matrix composition contributes to the structural development of the keel bone. Radiography of the whole keel has been proposed as a useful method for assessing keel bone damage and fractures (Eusemann et al., 2018), but no significant correlations were found between whole keel radiographic density and any of the composition traits in this study. This suggests that radiographic keel density captures structural and geometric aspects of the keel morphology that may be more critical for resisting mechanical loading (Li et al., 2024) and fractures (Maidin et al., 2024; Sallam et al., 2024a) than its compositional makeup.

We observed positive correlations between cranial cortical carbonate metrics (CO_3_1415/Amide I, MinCO_3_870) and keel length. Because carbonate substitution is a recognized feature of bone mineral that varies with tissue age or remodeling (Dominguez-Gasca et al., 2019) and relates to structural performance, these findings are compatible with regional changes in maturation of the cranial keel and its geometry, rather than competition for mineral resources.

Regarding the caudal part of the keel, a degree of mineralization (PO4/Amide I) in the keel cortex were positively correlated with keel length and may also contribute to the structural elongation of the keel. The positive correlation with the mineral-to-organic ratio (*r* = 0.30), indicates that greater cranial depth is associated with a compositional shift in the cranial cortex toward increased mineralization relative to the organic matrix. Crosslinking of collagen (or collagen maturity), as indicated by the LNK 1660/1690 ratio, in the caudal keel cortex was found to be associated with keel mid-depth (*r* = –0.48) or length-to-mid-depth ratio (*r* = 0.55), and cranial depth (*r* = –0.35), suggesting that changes in collagen crosslinking in the cortex may be linked to the development of thinner or more elongated keel structures or changes during growth. As it was previously shown in the literature, that maturity of collagen cross-linking may be responsible for the improved bone quality (De Koning et al., 2020), and is known to be correlated with osteoporosis in hens (Knott et al., 1995).

### Association between keel damage severity and keel bone composition

The current study demonstrated the differences in keel bone composition for different levels of severity of keel bone damage. Bone composition was assessed in both cranial and caudal regions of the keel across cortical and medullary compartments, but the significant compositional differences were only observed in the cortical bone of the cranial keel when correlated with keel damage measurements. Notably, lipid content in this region was significantly lower in birds with more extensive callus formation, covering one-third to two-thirds of the keel length, compared with birds showing no or minimal callus. In the context of keel bone health, a bigger callus generally indicates more extensive damage that requires greater bone remodeling (Baur et al., 2020; Sallam et al., 2024a). Reduced lipid content may be associated with changes in bone metabolic activity, potentially reflecting increased cellular turnover during remodeling in response to structural damage (Han et al., 2024). Similarly, Sallam et al. (2023) reported a negative correlation (*r* = –0.32) between the cortical lipid content and breaking strength recorded in tibia bone in Rhode Island Red laying hens. However, the mechanistic link between lipid depletion and bone mechanical properties remains to be fully elucidated.

In the cranial cortical bone, birds with moderate to severe callus formation (callus size) exhibited significantly lower mineral content and mineral-to-organic ratio, accompanied by a relative increase in organic content (Dunn et al., 2021; Sallam et al., 2023). This pattern reflects a shift toward reduced cortical mineralization, which is indicative of reduced keel bone quality and increased susceptibility to deformation and fracture (Gretarsson et al., 2024; Li et al., 2024; Toscano et al., 2020). Consistent with this interpretation, studies in Swiss laying hens reported that birds with intact or only mildly altered keel bones exhibited higher mineral content compared to those with fractures (Gebhardt-Henrich et al., 2017).

Notably, in our study, the most significant associations between callus formation (callus extent and size) with bone composition were observed in the cortex of the keel rather than in the medulla. This is consistent with the fact that external callus typically forms on the cortical surface at sites of damage. Moreover, the keel is a flat bone that is predominantly composed of cortical bone, with a comparatively limited medullary compartment, in contrast to long bones. Consequently, compositional changes associated with callus formation are expected to be most pronounced within the cortex of the keel.

Other observed changes in keel bone composition and their associations with keel damage or structural organization (e.g., CO_3_1415/AmideI vs. keel fracture count, collagen cross-linking vs. keel length) showed weak associations (*r* < 0.27). These results suggest that keel bone damage is likely driven by a complex interaction of physiological, genetic, environmental, and behavioral factors. Therefore, while composition play a role, the precise extent to which structural organization (e.g., collagen orientation) contributes to damage susceptibility need further investigation.

### Association between keel and tibia compositional traits

In the present study we demonstrated the relationship between biochemical composition of the keel and tibia bones. We found significant associations; however, the correlation coefficients were of modest magnitude, indicating that although compositional characteristics of the two bones are related, the strength of these relationships is relatively weak to moderate (Figs. S1, S2). These findings align with previous research (Benavides-Reyes et al., 2021; Sallam et al., 2025a), revealing that keel and tibia (tibiotarsus) bones exhibit some systematic differences in mineralization and composition. Thus, genomic selection for only tibia traits (Sallam et al., 2025b) may be insufficient to improve keel bone quality.

## Conclusion

This study provides clear evidence of regional differences in keel bone composition between the cranial and caudal keel. The cranial keel in its cortical bone exhibited greater collagen cross-linking (collagen maturity) and a more mineral-rich composition compared to the caudal region. Moreover, significant associations were observed between keel morphology and its biochemical composition. Importantly, increasing severity of keel damage (e.g., fractures in the caudal region, while deviations in the mid-keel) was linked to significant reductions in lipid content, mineral content, and the mineral-to-organic ratio in the cranial cortex. Together, these findings improve our understanding of keel bone biology in laying hens and highlight compositional factors that may underlie susceptibility to fractures. This knowledge may be useful for future research on genetic selection strategies and management practices aimed at improving keel bone health and welfare in commercial flocks.

## CRediT authorship contribution statement

**Khrystyna Kurta:** Writing – original draft, Formal analysis, Data curation. **Moh Sallam:** Writing – review & editing, Methodology, Investigation. **Lina Elmersson:** Writing – review & editing, Methodology, Investigation. **Tattiana Enrich-Essvein:** Methodology, Investigation. **Sky Vias-Valera:** Writing – review & editing, Methodology, Investigation. **Stefan Gunnarsson:** Writing – review & editing. **Helena Wall:** Writing – review & editing. **Ian C. Dunn:** Writing – review & editing. **Dirk-Jan de Koning:** Writing – review & editing, Resources, Project administration. **Martin Johnsson:** Writing – review & editing, Supervision, Resources, Project administration. **Alejandro B. Rodriguez-Navarro:** Writing – review & editing, Validation, Supervision, Resources, Project administration, Conceptualization.

## Disclosures

The authors declare that they have no known competing financial interests or personal relationships that could have appeared to influence the work reported in the present study.

## Appendix Supplementary materials

Image, application 1

## Footnotes

Supplementary material associated with this article can be found, in the online version, at doi:10.1016/j.psj.2026.106859.
