# Supplementary material for: Variation in cranial and caudal keel bone composition and its association with keel damage severity in laying hens
Source: Poult Sci. 2026 Mar 24;105(6):106859. doi: 10.1016/j.psj.2026.106859 (PMC13049312; doi:10.1016/j.psj.2026.106859)
Supplement: Supplementary file 1 [file mmc1.docx]

**Supplementary material**

Table S1. Summary statistics (number of samples (N) after removing missing values, median, mean, standard deviation, minimum and maximum) for keel bone composition traits.

| Trait | Side | Bone part | n | Median | Mean | SD | Min | Max |
| --- | --- | --- | --- | --- | --- | --- | --- | --- |
| **TGA** | | | | | | | | |
| Mineral/Organic | Caudal | cortex | 218 | 1.12 | 1.13 | 0.26 | 0.49 | 2.07 |
| Carbonate content | Caudal | cortex | 218 | 1.15 | 1.13 | 0.46 | 0 | 2.29 |
| Carbonate/Mineral | Caudal | cortex | 218 | 0.03 | 0.02 | 0.01 | 0 | 0.05 |
| Organic content | Caudal | cortex | 218 | 41.01 | 41.4 | 5.34 | 28.37 | 60.11 |
| Water content | Caudal | cortex | 219 | 12.79 | 13.1 | 1.57 | 10.29 | 18.25 |
| Mineral content | Caudal | cortex | 218 | 45.9 | 45.5 | 5.14 | 29.56 | 58.76 |
| Mineral/Organic | Cranial | cortex | 219 | 1.64 | 1.6 | 0.28 | 0.85 | 2.39 |
| Carbonate content | Cranial | cortex | 219 | 1.58 | 1.49 | 0.46 | 0 | 2.8 |
| Carbonate/Mineral | Cranial | cortex | 219 | 0.03 | 0.03 | 0.01 | 0 | 0.05 |
| Organic content | Cranial | cortex | 219 | 33.03 | 34.05 | 4.16 | 25.67 | 47.73 |
| Water content | Cranial | cortex | 219 | 12.35 | 12.54 | 1.92 | 8.23 | 34.71 |
| Mineral content | Cranial | cortex | 219 | 54.3 | 53.41 | 4.01 | 39.62 | 62.56 |
| **FTIR** | | | | | | | | |
| CO_3_ 1415/AmideI | Caudal | cortex | 214 | 0.23 | 0.24 | 0.09 | 0.07 | 0.55 |
| CO_3_ 1415/AmideI | Caudal | medulla | 214 | 0.16 | 0.18 | 0.07 | 0.07 | 0.69 |
| LNK 1660/1690 | Caudal | cortex | 214 | 4.91 | 5.08 | 1.49 | 1.86 | 13.2 |
| LNK 1660/1690 | Caudal | medulla | 214 | 3.66 | 3.7 | 0.7 | 1.85 | 9.67 |
| MinCO_3_1415 | Caudal | cortex | 213 | 0.25 | 0.38 | 0.44 | 0.03 | 4.03 |
| MinCO_3_1415 | Caudal | medulla | 188 | 0.6 | 3.53 | 13.49 | 0.1 | 148.74 |
| MinCO_3_870 | Caudal | cortex | 213 | 0.02 | 0.02 | 0.02 | 0 | 0.06 |
| MinCO_3_870 | Caudal | medulla | 188 | 0 | 0.01 | 0.01 | 0 | 0.07 |
| PO_4_/AmideI | Caudal | cortex | 214 | 0.96 | 1.06 | 0.75 | 0 | 4.39 |
| PO_4_/AmideI | Caudal | medulla | 214 | 0.22 | 0.43 | 0.49 | 0 | 2.2 |
| AmideI/AmideII | Caudal | cortex | 214 | 1.6 | 1.65 | 0.31 | 1.16 | 2.93 |
| AmideI/AmideII | Caudal | medulla | 214 | 1.52 | 1.54 | 0.18 | 0.75 | 2.59 |
| Lipids | Caudal | cortex | 214 | 0.04 | 0.05 | 0.08 | -0.12 | 0.64 |
| Lipids | Caudal | medulla | 214 | 0.08 | 0.11 | 0.09 | -0.1 | 0.42 |
| CO_3_ 1415/AmideI | Cranial | cortex | 217 | 0.41 | 0.4 | 0.11 | 0.14 | 0.61 |
| CO_3_ 1415/AmideI | Cranial | medulla | 219 | 0.16 | 0.17 | 0.07 | 0.07 | 0.7 |
| LNK 1660/1690 | Cranial | cortex | 216 | 5.69 | 6.34 | 3.13 | 1.93 | 23.36 |
| LNK 1660/1690 | Cranial | medulla | 219 | 3.81 | 3.78 | 0.53 | 1.91 | 5.2 |
| MinCO_3_1415 | Cranial | cortex | 217 | 0.18 | 0.21 | 0.17 | 0.11 | 2.39 |
| MinCO_3_1415 | Cranial | medulla | 204 | 0.46 | 1.24 | 2.8 | 0.05 | 30.62 |
| MinCO_3_870 | Cranial | cortex | 217 | 0.05 | 0.04 | 0.02 | 0 | 0.2 |
| MinCO_3_870 | Cranial | medulla | 204 | 0 | 0.01 | 0.01 | 0 | 0.05 |
| PO_4_/AmideI | Cranial | cortex | 217 | 2.26 | 2.34 | 1.1 | 0.06 | 4.78 |
| PO_4_/AmideI | Cranial | medulla | 219 | 0.35 | 0.49 | 0.54 | 0 | 3.03 |
| AmideI/AmideII | Cranial | cortex | 217 | 1.55 | 1.58 | 0.25 | 1.04 | 2.4 |
| AmideI/AmideII | Cranial | medulla | 219 | 1.57 | 1.6 | 0.2 | 0.79 | 2.31 |
| Lipids | Cranial | cortex | 217 | 0.04 | 0.05 | 0.06 | -0.07 | 0.3 |
| Lipids | Cranial | medulla | 219 | 0.09 | 0.12 | 0.12 | -0.07 | 0.62 |

Table S2. Summary statistics (number of samples (N) after removing missing values, median, mean, standard deviation, minimum and maximum) for tibia bone composition traits averaged per left and right bones.

| Trait | Bone part | N | Median | Mean | SD | Min | Bone part |
| --- | --- | --- | --- | --- | --- | --- | --- |
| **TGA** | | | | | | | |
| Mineral/Organic | cortex | 218 | 2.82 | 2.79 | 0.2 | 1.95 | 3.29 |
| Mineral/Organic | medulla | 218 | 0.64 | 0.67 | 0.36 | 0.08 | 2.72 |
| Carbonate content | cortex | 218 | 1.29 | 1.35 | 0.47 | 0.32 | 2.64 |
| Carbonate content | medulla | 218 | 0.69 | 0.75 | 0.49 | -0.19 | 2.43 |
| Carbonate/Mineral | cortex | 218 | 0.02 | 0.02 | 0.01 | 0.01 | 0.04 |
| Carbonate/Mineral | medulla | 218 | 0.02 | 0.02 | 0.02 | -0.02 | 0.22 |
| Organic content | cortex | 218 | 23.16 | 23.38 | 1.45 | 20.61 | 30.03 |
| Organic content | medulla | 218 | 53.78 | 55.11 | 10.79 | 23.75 | 86.23 |
| Water content | cortex | 218 | 11.98 | 11.81 | 1.25 | 8.5 | 16.33 |
| Water content | medulla | 218 | 12.07 | 12.17 | 1.84 | 3.42 | 26.19 |
| Mineral content | cortex | 218 | 65.03 | 64.82 | 1.43 | 57.15 | 67.44 |
| Mineral content | medulla | 218 | 33.68 | 32.73 | 10.11 | 6.49 | 63.57 |
| **FTIR** | | | | | | | |
| CO_3_ 1415/AmideI | cortex | 218 | 1.04 | 1.04 | 0.12 | 0.71 | 1.6 |
| CO_3_ 1415/AmideI | medulla | 217 | 0.25 | 0.27 | 0.08 | 0.13 | 0.93 |
| LNK 1660/1690 | cortex | 218 | 4.57 | 5.67 | 5.03 | 2.03 | 68.64 |
| LNK 1660/1690 | medulla | 217 | 3.37 | 3.37 | 0.24 | 2.64 | 4.22 |
| MinCO_3_1415 | cortex | 218 | 0.21 | 0.21 | 0.02 | 0.15 | 0.35 |
| MinCO_3_1415 | medulla | 212 | 1.58 | 14.23 | 55.61 | 0.28 | 539.08 |
| MinCO_3_870 | cortex | 218 | 0.08 | 0.08 | 0.01 | 0.05 | 0.09 |
| MinCO_3_870 | medulla | 212 | 0 | 0 | 0.01 | 0 | 0.04 |
| PO_4_/AmideI | cortex | 218 | 4.99 | 5.07 | 0.93 | 2.67 | 9.12 |
| PO_4_/AmideI | medulla | 217 | 0.2 | 0.33 | 0.36 | 0 | 1.83 |
| AmideI/AmideII | cortex | 218 | 1.11 | 1.12 | 0.1 | 0.86 | 1.96 |
| AmideI/AmideII | medulla | 217 | 1.28 | 1.31 | 0.17 | 0.7 | 2.34 |
| Lipids | cortex | 218 | 0.01 | 0.02 | 0.02 | 0 | 0.12 |
| Lipids | medulla | 217 | 0.04 | 0.04 | 0.04 | -0.12 | 0.28 |

Table S3. Summary statistics (median, mean, and standard deviation) for keel bone composition traits by keel damage category.

| Bone part | Method | Keel composition trait | Keel damage trait | Category | Median | Mean | SD |
| --- | --- | --- | --- | --- | --- | --- | --- |
| Cranial cortex | TGA | Organic content | Callus size | 0: no callus | 32.67 | 33 | 2.63 |
| Cranial cortex | TGA | Organic content | Callus size | 1: minimum callus | 31.65 | 33.28 | 4.13 |
| Cranial cortex | TGA | Organic content | Callus size | 2: moderate to severe callus | 36.23 | 36.39 | 5.66 |
| Cranial cortex | TGA | Mineral content | Callus size | 0: no callus | 55.07 | 54.83 | 2.45 |
| Cranial cortex | TGA | Mineral content | Callus size | 1: minimum callus | 55.81 | 54.37 | 3.95 |
| Cranial cortex | TGA | Mineral content | Callus size | 2: moderate to severe callus | 52.12 | 51.37 | 5.24 |
| Cranial cortex | TGA | Mineral/Organic | Callus size | 0: no callus | 1.7 | 1.67 | 0.19 |
| Cranial cortex | TGA | Mineral/Organic | Callus size | 1: minimum callus | 1.79 | 1.67 | 0.3 |
| Cranial cortex | TGA | Mineral/Organic | Callus size | 2: moderate to severe callus | 1.43 | 1.46 | 0.35 |
| Caudal cortex | TGA | Mineral content | Deviation size | 0: no deviation | 47.77 | 47.93 | 3.49 |
| Caudal cortex | TGA | Mineral content | Deviation size | 1: caudal only | 47.61 | 48.15 | 2.86 |
| Caudal cortex | TGA | Mineral content | Deviation size | 2: middle only | 46.5 | 44.85 | 5.67 |
| Cranial cortex | FTIR | Lipids | Callus size | 0: no callus | 0.06 | 0.07 | 0.03 |
| Cranial cortex | FTIR | Lipids | Callus size | 1: minimum callus | 0.03 | 0.04 | 0.04 |
| Cranial cortex | FTIR | Lipids | Callus size | 2: moderate to severe callus | 0.03 | 0.04 | 0.05 |
| Cranial cortex | FTIR | Lipids | Callus extent | 0: no callus | 0.07 | 0.07 | 0.03 |
| Cranial cortex | FTIR | Lipids | Callus extent | 1: callus in one third of keel | 0.03 | 0.04 | 0.04 |
| Cranial cortex | FTIR | Lipids | Callus extent | 2: callus in two thirds of keel | 0.03 | 0.02 | 0.02 |
| Cranial cortex | FTIR | AmideI/AmideII | Fractures count | 0: no fractures | 1.59 | 1.57 | 0.17 |
| Cranial cortex | FTIR | AmideI/AmideII | Fractures count | 1: one fracture | 1.51 | 1.59 | 0.32 |
| Cranial cortex | FTIR | AmideI/AmideII | Fractures count | 2: two fractures | 1.65 | 1.63 | 0.26 |
| Cranial cortex | FTIR | AmideI/AmideII | Fractures count | 3: three fractures | 1.44 | 1.45 | 0.09 |
| Cranial cortex | FTIR | AmideI/AmideII | Fractures count | 4: >four fractures | 1.45 | 1.44 | 0.13 |

Figure S1. Pearson correlations between keel bone and tibia composition traits measured with TGA. Statistical significance is indicated as follows: **P* < 0.05, ***P* < 0.01, ****P* < 0.001.

Figure S2. Pearson correlations between keel bone and tibia composition traits measured with FTIR. Statistical significance is indicated as follows: **P* < 0.05, ***P* < 0.01, ****P* < 0.001.
